# Utilising a multi‐item questionnaire to assess household food security in Australia

**DOI:** 10.1002/hpja.61

**Published:** 2018-04-11

**Authors:** Lucy M. Butcher, Therese A. O'Sullivan, Maria M. Ryan, Johnny Lo, Amanda Devine

**Affiliations:** ^1^ Edith Cowan University Joondalup WA Australia; ^2^ Foodbank WA Perth Airport WA Australia

**Keywords:** food insecurity, food poverty, food security, health policy, nutrition, public policy, quantitative methods

## Abstract

**Issue addressed:**

Currently, two food sufficiency questions are utilised as a proxy measure of national food security status in Australia. These questions do not capture all dimensions of food security and have been attributed to underreporting of the problem. The purpose of this study was to investigate food security using the short form of the US Household Food Security Survey Module (HFSSM) within an Australian context; and explore the relationship between food security status and multiple socio‐demographic variables.

**Methods:**

Two online surveys were completed by 2334 Australian participants from November 2014 to February 2015. Surveys contained the short form of the HFSSM and twelve socio‐demographic questions. Cross‐tabulations chi‐square tests and a multinomial logistic regression model were employed to analyse the survey data.

**Results:**

Food security status of the respondents was classified accordingly: *High or Marginal* (64%, n = 1495), *Low* (20%, n = 460) or *Very Low* (16%, n = 379). Significant independent predictors of food security were age (*P *<* *0.001), marital status (*P *=* *0.005), household income (*P *<* *0.001) and education (*P *<* *0.001).

**Conclusion:**

Findings suggest food insecurity is an important issue across Australia and that certain groups, regardless of income, are particularly vulnerable.

**So what?:**

Government policy and health promotion interventions that specifically target “at risk” groups may assist to more effectively address the problem. Additionally, the use of a multi‐item measure is worth considering as a national indicator of food security in Australia.

## 1 INTRODUCTION

Food insecurity (FI) is considered by many to be a problem specific to developing nations. Nevertheless, it is also prevalent within the developed nation of Australian, albeit more subtle. An estimated four per cent of Australians do not have sufficient food to eat, despite Australia producing twice as much food as it consumes.^1^, ^2^ The internationally accepted definition of food security is “when all people, at all times, have physical, social and economic access to sufficient, safe and nutritious food that meets their dietary needs and food preferences for an active and healthy life”.^3^ Sensitive and accurate measurement of food security along with identification of country‐specific risk factors is vital for the provision of targeted support services. In addition, a precise understanding of the nature and size of the problem may assist in driving government policy direction, social service provision and evaluation of the impact of food insecurity.

Food sufficiency and food security are often used interchangeably in the literature. Specifically, food sufficiency is a physical concept, referring to the adequate intake of food to meet requirements.^4^ Food security (FS) is a broader concept and is inclusive of food sufficiency, but also acknowledges psychological, social and cultural factors.^4^ The Australian Bureau of Statistics (ABS) utilises food sufficiency questions, rather than a dedicated FS instrument, as a proxy to monitor FS nationally.^5^ Prior to 2011, the National Nutrition Survey used a single question to estimate the proportion of the population that was food insecure: “In the past 12 months, have you or anyone in your household run out of food and not had enough money to purchase more?”.^5^ The 2011‐2012 National Nutrition and Physical Activity Survey used a similarly worded question and an additional question. The survey read: “In the past 12 months was there any time when you or members of your household ran out of food and couldn't afford to buy more?” and “When this happened, did you or members of your household go without food?”.^6^ Responses to food sufficiency questions define individuals as food sufficient or insufficient; however, they do not reveal the severity of insufficiency.

The failure of ABS food sufficiency questions to capture the full spectrum of FI has been attributed to underreporting of the problem in Australia.^7^ These questions measure food deprivation, which only encompasses the severest form of FI. The focus of this type of FS measure is food shortage due to financial constraints, and more diverse contributing factors such as social and psychological dimensions are missed.^8^ Mild‐to‐moderate FI is often overlooked by food sufficiency questions meaning the true extent of the problem cannot be ascertained.^8^ Food insufficiency can be interpreted as a proxy for “FI with hunger” or “Very Low” FS.^8^

An alternative FS surveillance instrument to the ones commonly used in Australia is the 18‐item questionnaire Household Food Security Survey Module (HFSSM).^9^ The HFSSM is administered annually in the United States, and adaptations of this instrument have been used in several other countries, such as Canada, Mexico and Brazil. Changes in reported food intake, as a consequence of declining household resources, and the severity of FI are indicated by the HFSSM. These aspects are missed by food sufficiency questions.^9^ Validation of the HFSSM involved 44 647 household interviews derived from the April 1995 U.S. Current Population Survey.^10^ A more concise six‐item version of the HFSSM was developed in 1999 using data from the same survey. The short form questionnaire has comparable accuracy to the longer form (it correctly identified 97.7% of households as food secure), whilst having the advantage of reduced respondent burden.^11^

It is widely accepted in Australia and internationally that there is interplay between socio‐demographic characteristics and FS status.^12^, ^13^ Income has been prolifically studied and is often considered the most significant factor in predicting FS.^12^, ^13^, ^14^ However, many other demographic characteristics, such as age, gender, marital status and education, may have an additional impact, above and beyond, income.^12^, ^15^, ^16^

To our knowledge, no previous studies have utilised a standardised multi‐item FS instrument and reviewed multiple socio‐demographic factors at a general population level in multiple Australian states. Therefore, the purposes of this study were to investigate FS using the short form of the HFSSM within an Australian sample population; and explore the relationship between FS status and the multiple social, economic and physical dimensions encapsulated within the definition of FS.

## 2 METHODS

As part of a larger multiproject food consumption behaviours research program, two online surveys were administered from November 2014 to February 2015 to 2334 Australian participants from the general population. Survey one (n = 1056) assessed shopping, food choice, cooking and consumption behaviours. Survey two (n = 1278) investigated eating away from home practices, such as shopping centre food court and restaurant consumption, and food advertising. The primary content of each survey varied; however, both surveys contained the short form of the United States. HFSSM and a number of socio‐demographic measures including age, gender, immigration, occupation, education, household income, household structure and marital status. The six‐item short form of the HFSSM (Table 2) was utilised to reduce respondent burden.

### 2.1 Selection and description of participants

The surveys were administered through Qualtrics (Provo, Utah, USA) and disseminated online to registered panel respondents through a commercial research marketing company. Inclusion criteria were that the respondent was the main grocery purchaser in the household, resided in one of five states of Australia (New South Wales (NSW), Victoria (VIC), Western Australia (WA), South Australia (SA) and Queensland (QLD)), was aged between 18 and 84 years, and had computer ownership or access. No additional exclusion criteria were applied. Quotas were set to ensure sufficient representation terms of gender, age groups and location. A minimum proportion of male representation was set at 30%. The quotas for were set to align with national Australian population demographics for age: 10% were aged 19‐24 years; 20% 25‐34 years; 19% 35‐44 years; 18% 45‐54 years; 15% 55‐64 years; 18% 65‐84 years, and for location: 34% NSW; 26% VIC; 11% WA; 7% SA; 22% QLD.^17^ De‐identified data were available for analysis.

### 2.2 Statistics

Survey data were cleaned and analysed using Statistical Package for Social Sciences (SPSS) (IBM Corp. Armonk, NY, USA). Several of the socio‐demographic variables were recoded due to low cell counts and for ease of analysis. Recoded variables included number of children and adults in the household, income, occupation, year of arrival and highest level of education attained. Occupation status comprised 45 options, which was collapsed into the eight major groups of occupations as defined by the ABS.^18^ Year of arrival in Australia was regrouped into periods of immigration (White Australia policy 1900‐1944, post‐second world war 1945‐1975, Indochinese immigration 1976‐1999 and modern immigration 2000‐2013) as set out by the Australian Department of Immigration and Border Protection.^19^

Household income was reduced from 13 categories to six and based on the 2016/2017 Australian Tax Office income brackets. The categories for household income are as follows: refused to answer, very low (<$18 000), low ($18 001‐37 000), middle ($37 001‐87 000), high ($87 001‐180 000) and very high (>$180 000).^20^ The number of children (classified as <18 years of age) residing in the household variable was reduced from seven to four categories (0, 1, 2 and 3 or more). Likewise, the number of adults in the household was reduced from seven to three categories (1, 2 and 3 or more). Highest education status achieved was recoded as either secondary or less, vocational or university and based on the ABS categories.^21^

Responses to the six HFSSM questions **(**Q1‐Q6, see Table 2) were coded and assessed in accordance with the US HFSSM user notes.^22^ In brief, any affirmative responses, including “yes,” “sometimes true” and “often true,” were assigned a score of one. The sum of the affirmative responses (range 0‐6) from the six questions was calculated to classify the household into three levels of FS:

Score 0‐1: *High or Marginal* FS, Score 2‐4: *Low* FS, and Score 5‐6: *Very Low* FS.

Cross‐tabulations and chi‐square tests were initially employed to explore the relationship between FS status and^1^ responses to each of the six HFSSM questions, and^2^ with each of the socio‐demographic variables. The socio‐demographic variables that were individually significantly associated with FS status were then entered into a multinomial logistic regression model to formally examine their relationship with FS status. The *High or Marginal* FS respondents were utilised as the reference group in this model, and statistical significance was set at a *P *≤* *0.05. Descriptive statistics in the form of frequency (%) are presented.

### 2.3 Ethics approval

All procedures performed in studies involving human participants were in accordance with the ethical standards of the institutional and/or national research committee and with the 1964 Helsinki Declaration and its later amendments or comparable ethical standards. Informed consent was obtained from all individual participants included in the study. Ethics approval has been granted by Edith Cowan University's Human Research Ethics Committee.

## 3 RESULTS

### 3.1 Sample demographics

Characteristics of the survey respondents are given in Table 1. In terms of income, the greatest representation was in the low bracket ($18 001‐37 000, 31%) followed by middle ($37 001‐87 000) and high ($87 001‐180 000) income earners equally (24%). Approximately 3 in 5 (61%) of the respondents were married or in a de facto relationship. Almost three‐quarters of the respondents (73%) had attained some form of post‐secondary education (Table 1). The distributions across the age groups were as follows: 19‐24: 11%, 25‐34: 19%, 35‐44: 19%, 45‐54: 19%, 55‐64: 17%, 65‐84: 15% and locations were as follows: NSW 34%, VIC 27%, WA 11%, SA 7%, and QLD 21%.

**Table 1:** Characteristics of survey respondents and individual demographic variable association with food security status

Independent variable	Category	High‐marginal food security (%)	Low food security (%)	Very low food security (%)	Total n (% of N = 2334) (%)	Individual association with food security status
Age (y)	19‐24	141 (52.8)	76 (28.5)	50 (18.7)	267 (11.4)	<0.001
	25‐ 34	242 (54.3)	114 (25.6)	90 (20.2)	446 (19.1)	
	35‐44	246 (57.1)	96 (22.3)	89 (20.6)	431 (18.5)	
	45‐54	298 (67.1)	77 (17.3)	69 (15.5)	444 (19.0)	
	55‐64	278 (71.5)	60 (15.4)	51 (13.1)	389 (16.7)	
	65‐84	290 (81.2)	37 (10.4)	30 (8.4)	357 (15.3)	
Marital status	Widowed	55 (78.6)	7 (10.0)	8 (11.4)	70 (3.0)	<0.001
	Divorced/Separated	140 (57.4)	42 (17.2)	62 (25.4)	244 (10.5)	
	Married/Defacto	959 (67.6)	259 (18.3)	201 (14.2)	1419 (60.8)	
	Single	341 (56.7)	152 (25.3)	108 (18.0)	601 (25.7)	
Number of adults in the household	1	251 (58.0)	91 (21.0)	91 (21.0)	433 (18.6)	0.009
	2	891 (65.9)	251 (18.6)	211 (15.6)	1353 (58.0)	
	3 or more	353 (64.4)	118 (21.5)	77 (14.1)	548 (23.5)	
Number of children (>18 y) in the household	0	1091 (67.1)	291 (17.9)	245 (15.1)	1627 (69.7)	<0.001
	1	197 (56.9)	85 (24.6)	64 (18.5)	346 (14.8)	
	2	150 (60.7)	48 (19.4)	49 (19.8)	247 (10.6)	
	3 or more	57 (50.0)	36 (31.6)	21 (18.4)	114 (4.9)	
Household income	Refused to answer	145 (67.4)	44 (20.5)	26 (12.1)	215 (9.2)	<0.001
	Very low (<$18 000)	29 (39.2)	21 (28.4)	24 (32.4)	74 (3.2)	
	Low ($18 001‐37 000)	417 (57.4)	153 (21)	157 (21.6)	727 (31.1)	
	Middle ($37 001‐87 000)	365 (64.6)	107 (18.9)	93 (16.5)	565 (24.2)	
	High ($87 001‐180 000)	389 (70)	105 (18.9)	62 (11.2)	556 (23.8)	
	Very high (>$180 000)	150 (76.1)	30 (15.2)	17 (8.6)	197 (8.4)	
Education	Secondary or less	367 (59.1)	140 (22.5)	114 (18.4)	621 (26.6)	<0.001
	Vocational	568 (62)	185 (20.2)	163 (17.8)	916 (39.2)	
	University	560 (70.3)	135 (16.9)	102 (12.8)	797 (34.1)	
Occupation	Managers	107 (60.8)	40 (22.7)	29 (16.5)	176 (7.5)	<0.001
	Professionals	348 (64.9)	111 (20.7)	77 (14.4)	536 (23.0)	
	Technicians and Trades workers	87 (53.7)	40 (24.7)	35 (21.6)	162 (6.9)	
	Community and Personal Service	102 (64.6)	23 (14.6)	33 (20.9)	158 (6.8)	
	Clerical/Administrative Workers	211 (63.4)	68 (20.4)	54 (16.2)	333 (14.3)	
	Sales Workers	85 (58.6)	39 (26.9)	21 (14.5)	145 (6.2)	
	Machinery Operators and Drivers	23 (67.6)	7 (20.6)	4 (11.8)	34 (1.5)	
	Labourers	109 (50.5)	52 (24.1)	55 (25.5)	216 (9.3)	
	Retired	423 (73.7)	80 (13.9)	71 (12.4)	574 (24.6)	
Immigration	Post‐WW2 (1945‐1975)	140 (74.1)	26 (13.8)	23 (12.2)	189 (8.1)	0.012
	Indochinese (1976‐ 1999)	130 (68.4)	29 (15.3)	31 (16.3)	190 (8.1)	
	Modern migration (2000+)	132 (61.4)	54 (25.1)	29 (13.5)	215 (9.2)	
	Australian born	1093 (62.8)	351 (20.2)	296 (17.0)	1740 (74.6)	

### 3.2 Distribution of HFSSM responses across FS status

Food security status of the respondents was either *High or Marginal* (64% n = 1495), *Low* (20%, n = 460) or *Very Low* (16%, n = 379). Those classified as having *Low* or *Very Low* FS were most likely to respond in the affirmative to the six questions (Table 2). For instance, 76% and 97% of those in the *Low* and *Very Low* FS classifications gave an affirmative response to Q1: “The food that (I/we) bought just didn't last, and (I/we) didn't have money to get more,” with almost 2 in 5 (37%) in the latter category indicating that the situation is often true for them. In contrast, less than 1 in 10 (7.3%) of those with *High or Marginal* FS, considered the above as an issue. This pattern of response continued for the remaining HFSSM questions.

**Table 2:** Responses to the Household Food Security Survey Module by food security category

Household food security survey module questions		High‐ marginal food security (n = 1495) (%)	Low food security (n = 460) (%)	Very low food security (n = 379) (%)
Q1—“The food that (I/we) bought just didn't last, and (I/we) didn't have money to get more.” Was that often, sometimes, or never true for (you/your household) in the last 12 months?	Never true	1313 (87.8)	85 (18.5)	10 (2.6)
	Sometimes true	102 (6.8)	284 (61.7)	227 (59.9)
	Often true	7 (0.5)	66 (14.3)	141 (37.2)
	Don't know or Refused	73 (4.9)	25 (5.4)	1 (0.3)
Q2—“(I/we) couldn't afford to eat balanced meals.” Was that often, sometimes, or never true for (you/your household) in the last 12 months?	Never true	1339 (89.6)	107 (23.3)	23 (6.1)
	Sometimes true	77 (5.2)	250 (54.3)	200 (52.8)
	Often true	16 (1.1)	75 (16.3)	154 (40.6)
	Don't know or Refused	63 (4.2)	28 (6.1)	2 (0.5)
Q3—In the last 12 months, did (you/you or other adults in your household) ever cut the size of your meals or skip meals because there wasn't enough money for food?	Yes	0 (0.0)	212 (46.1)	379 (100.0)
	No	1495 (100.0)	248 (53.9)	0 (0.0)
Q4—[IF YES ABOVE] How often did this happen—almost every month, some months but not every month, or in only 1 or 2 months?	Almost every month	0 (0)	28 (13.2)	120 (31.7)
	Some months but not every month	0 (0)	78 (36.8)	169 (44.6)
	Only 1 or 2 months	0 (0)	106 (50.0)	90 (23.7)
Q5—In the last 12 months, did you ever eat less than you felt you should because there wasn't enough money for food?	Yes	23 (1.5)	129 (28.0)	363 (95.8)
	No	1421 (95.1)	284 (61.7)	13 (3.4)
	Don't know	51 (3.4)	47 (10.2)	3 (0.8)
Q6—In the last 12 months, were you ever hungry but didn't eat because there wasn't enough money for food?	Yes	9 (0.6)	88 (19.1)	306 (80.7)
	No	1445 (96.7)	337 (73.3)	68 (17.9)
	Don't’ know	41 (2.7)	35 (7.6)	5 (1.3)

One particular question was polarising: Q3 “In the last 12 months, did (you/you or other adults in your household) ever cut the size of your meals or skip meals because there wasn't enough money for food?” All of the *High or Marginal* FS respondents disagreed with this question, whilst all of the *Very Low* FS respondents agreed with the statement.

Other notable disparities were observed in Q5 and Q6 where 96% and 81%, respectively, of those in the *Very Low* FS category responded “yes” to having to eat less or not eat at all because there was not enough money for food, whereas for those with *Low* FS, only 28% and 19%, respectively, responded in the same way. In comparison, over 95% of the *High or Marginal* FS respondents did not have to reduce their food consumption in the past 12 months due to insufficient funds.

### 3.3 Determinants of FS status

No significant association was observed individually between FS status and respondents’ gender (*P *=* *0.448), location (*P *=* *0.151) or being a first‐, second‐ or third‐generation migrant (*P *=* *0.346). Conversely, age, household income, marital status, number of adults and children in the household, occupation, immigration and highest level of education attainment were shown to have a significant association with FS status (Table 1). When analysed using a multivariable multinomial logistic regression model, the only significant independent predictors of increasing risk of FI were younger age (*P *<* *0.001), divorce or separation (*P *=* *0.005), lower household income (*P *<* *0.001) and lower educational attainment (*P *<* *0.001) (Table 3).

**Table 3:** Significant predictors of food security status as determined by the multivariable multinomial logistic regression model

Independent variable	Category	Overall	High‐marginal vs low food security		High‐marginal vs very low food security	
			OR (95% OR)	*P* ‐value	OR (95% OR)	*P* ‐value
Age (y)	19‐24	<0.001	4.52 (2.45, 8.34)	<0.001	6.58 (3.31, 13.05)	<0.001
	25‐ 34		4.81 (2.79, 8.32)	<0.001	7.69 (4.19, 14.11)	<0.001
	35‐44		3.98 (2.29, 6.92)	<0.001	6.98 (3.82, 12.73)	<0.001
	45‐54		2.33 (1.40, 3.86)	0.001	3.17 (1.83, 5.49)	<0.001
	55‐64		1.93 (1.21, 3.09)	0.006	2.11 (1.26, 3.53)	0.004
	65‐84		1.00 (ref)		1.00 (ref)	
Marital status	Widowed	0.005	0.52 (0.22, 1.23)	0.136	1.01 (0.43, 2.35)	0.988
	Divorced/Separated		1.00 (0.63, 1.59)	1.000	2.33 (1.48, 3.69)	<0.001
	Married/Defacto		0.97 (0.69, 1.38)	0.878	1.13 (0.77, 1.67)	0.530
	Single		1.00 (ref)		1.00 (ref)	
Number of adults in the household	1	0.736	1.19 (0.79, 1.79)	0.407	1.14 (0.73, 1.78)	0.560
	2		0.96 (0.71, 1.30)	0.807	1.16 (0.83, 1.63)	0.385
	3 or more		1.00 (ref)		1.00 (ref)	
Number of children (>18 y) in the household	0	0.174	1.00 (ref)		1.00 (ref)	
	1		1.30 (0.94, 1.79)	0.109	1.16 (0.81, 1.66)	0.417
	2		1.06 (0.72, 1.57)	0.765	1.14 (0.75, 1.72)	0.537
	3 or more		1.94 (1.20, 3.14)	0.007	1.17 (0.66, 2.09)	0.596
Household income	Refused to answer	<0.001	1.95 (1.14, 3.35)	0.015	2.06 (1.05, 4.04)	0.036
	Very low (<$18 000)		4.59 (2.19, 9.61)	<0.001	9.6 (4.34, 21.24)	<0.001
	Low ($18 001‐37 000)		3.06 (1.89, 4.97)	<0.001	5.40 (3.02, 9.65)	<0.001
	Middle ($37 001‐87 000)		1.70 (1.07, 2.70)	0.026	2.53 (1.44, 4.46)	0.001
	High ($87 001‐180 000)		1.36 (0.86, 2.14)	0.194	1.41 (0.79, 2.51)	0.248
	Very high (>$180 000)		1.00 (ref)		1.00 (ref)	
Education	Secondary or less	<0.001	1.89 (1.37, 2.62)	<0.001	1.73 (1.21, 2.48)	0.003
	Vocational		1.52 (1.14, 2.02)	0.004	1.46 (1.07, 2.01)	0.017
	University		1.00 (ref)		1.00 (ref)	
Occupation	Managers	0.249	1.43 (0.86, 2.38)	0.166	1.27 (0.72, 2.24)	0.407
	Professionals		1.32 (0.86, 2.03)	0.209	1.16 (0.73, 1.87)	0.530
	Technicians and Trades workers		1.72 (1.02, 2.89)	0.040	2.09 (1.20, 3.62)	0.009
	Community and Personal Service		0.80 (0.45, 1.42)	0.446	1.30 (0.75, 2.25)	0.347
	Clerical/Administrative Workers		1.13 (0.73, 1.76)	0.583	1.09 (0.68, 1.76)	0.716
	Sales Workers		1.38 (0.82, 2.32)	0.225	0.88 (0.48, 1.63)	0.694
	Machinery Operators and Drivers		1.18 (0.46, 2.99)	0.733	0.92 (0.29, 2.92)	0.893
	Labourers		1.15 (0.71, 1.86)	0.582	1.44 (0.87, 2.38)	0.151
	Retired		1.00 (ref)		1.00 (ref)	
Immigration	Post‐WW2 (1945‐1975)	0.559	1.06 (0.66, 1.70)	0.819	1 (0.6, 1.67)	0.987
	Indochinese (1976‐ 1999)		0.81 (0.52, 1.26)	0.354	0.98 (0.63, 1.52)	0.912
	Modern migration (2000+)		1.21 (0.83, 1.75)	0.318	0.75 (0.47, 1.18)	0.213
	Australian born		1.00 (ref)		1.00 (ref)	

The eldest group (65‐84 years) of respondents were least likely to have *Low* (10%) or *Very Low* (8%) FS status. In contrast to the eldest group, respondents aged 45‐64 years were 2‐3 times more likely to have *Low* or *Very Low* FS (*P *<* *0.001); those aged 44 years or younger were 4‐5 times more likely to experience Low FS and 6‐8 times more likely to be in the *Very Low* FS category (*P *<* *0.001).

Respondents in the lowest household income bracket (<$18 000) were, respectively, 5‐10 times more likely to be of *Low* or *Very Low* FS status than their very high household income (>$180 000) counterparts where 3 in 4 respondents were classified as having *High or Marginal* FS (Table 3).

Respondents who were divorced or separated were 2.3 times more likely to be categorised with *Very Low* FS status than those who were single (*P *<* *0.001, Table 3). Of those who reported their income and were not retired, 54% of respondents who were either divorced or separated were in the low‐ or very low‐income bracket, and only 14% were in the high‐ or very high‐income brackets. In comparison with those who were married (or in a de facto relationship), 17% were in the low‐ or very low‐income bracket and 54% were in the high‐ or very high‐income bracket. Respondents with secondary level education or less were 89% and 73% more likely to be categorised as having *Low* and *Vey Low* FS status, respectively, compared to their university‐educated counterparts, where 70% were deemed to have *High or Marginal* FS.

## 4 DISCUSSION

Greater than a third (36%) of the study population had some degree of FI (defined as *Low* or *Very Low* FS status), significantly higher than the 4% of food insufficiency reported by the ABS 2015. When other studies have applied a multi‐item instrument to a subset of the Australian population, they too have documented a disparity between their results and those reported by the ABS food sufficiency questions‐ implying the ABS measure underestimates cases of FI.^13^, ^23^ As previously indicated, the HFSSM multi‐item instrument has been validated and utilised extensively internationally. Whilst the reasoning behind the ongoing use of the ABS measure is unknown, it seems reasonable to suggest that the comparatively higher results using a specialised multi‐item tool may provide a more accurate indication of the extent of FI in the Australian population.

Similar to other studies,^12^, ^13^, ^16^, ^24^ we established that income was a strong predictor of FS status in this population and it remained after adjustment of age, marital status and education (also significant predictors of FS status). Affording sufficient quantities, especially foods of a high nutritional value, is a challenge and represents a large proportion of the budget, for those on a lower income.^16^, ^25^, ^26^ Nevertheless, our findings reveal that FI is not exclusive to low‐income households. Previous research has suggested that this problem also exists within higher income households.^24^, ^27^ Chronic health conditions, job losses or spending on gambling or tobacco can create financial instability and put strain on food budgets regardless of household income.^27^

The risk of FI generally appears to decrease with age in this study population. This is consistent with other research in the area, where FI risk starts to decline at age 45 and reaches its lowest rate at over 65 years of age.^12^, ^28^ Homeownership, government assistance and children leaving home may be explanations for a reduction of FI in the over 65 age group in this and other studies.^12^, ^28^ Older Australians are less likely to rent than their younger counterparts and this may account for the inconsistency in FS status between age groups. Residing in rental accommodation has been found to have a positive association with FI.^15^, ^29^, ^30^ Alternatively, several factors are recognised in the literature as heightening the risk of FI in the elderly, although not seen in this population, which include poor health, low income, reduced ability to drive or carry shopping, decreased appetite, and social isolation. One theory is that older people become accustomed to deprivation, thus chronic dietary compromises result in underreporting of the problem.^31^

Marital status also appears to be linked to FS. Divorced or separated people appear to be at greatest risk of FI, whilst being married or widowed are protective both in the findings of this study and others.^16^, ^32^ The division of income across households, the cost of child support and lack of social support have been suggested as causal mechanisms for the relationship between separation and FI.^32^

There is minimal research in developed nations investigating the relationship between education attainment and FS. University level education was shown to be a protective factor in our study. Thornton, Pearce^16^ suggested that greater education attainment may improve employability and the likelihood of achieving a higher income, which may ultimately reduce FI risk. Despite this, our findings suggest that there is an association between education attainment and FS status, independent of income and occupation. It is possible that greater educational attainment may improve food literacy, including budgeting skills, thus reducing the likelihood of being food insecure. However, this is in contrast to the present North American consensus that financial restraints prevent the uptake of healthy diet and limit the individual's ability to successfully employ food literacy skills.^33^ Further research is warranted in the Australian context to get a clearer depiction of the impact of food literacy skills on FS status.

### 4.1 Application of findings and limitations

The reference period of our study included the Christmas or festive season. All of the short form HFSSM questions, however, refer to “in the last 12 months” suggesting that the festive season should not have impacted the results. It is possible that respondents may have been experiencing higher than normal financial stress at this time, bringing the concept of deprivation to front of mind and potentially skewing the findings.

The study population was similar, but not identical to the Australian general population. Sixty per cent of the respondents were females, which was approximately 10% more than that of the general Australian population. The distributions across the age groups and locations of the study population were within 3% and 2%, respectively, of the general Australian population.^17^ Excluding those who refused to answer, the proportion of respondents in the very low‐income brackets (3.5%) was severely underrepresented when compared to the Australian population (18.8%); conversely, there was an overrepresentation of high (26%)‐ and very high (9.3%)‐income earners in the study population as compared to the Australian population (high 17.2%; very high 3%).^20^ The majority (61%) of the respondents in this study were married or in a de facto relationship, which is similar to the overall Australian population where 54% were married and 11% were in a de facto relationship.^6^ The post‐secondary educational attainment of the study population (73%) was 16% higher when compared to the overall Australian population (57%).^17^ As household income and education attainment are significant predictors of FS status, the noted small numbers of participants with low education attainment and very low household incomes in the study population may have skewed the results. The household structure (number of adults and children) was adjusted for in the multivariate model, but due to the study design equivalisation could not be employed. These factors could have potentially reduced the proportion of FI and lessen the applicability of the overall findings to the general Australian population. Nevertheless, our results still provide a valuable insight into the interplay between demographic variables and FS status in the Australian context.

Our study did not differentiate between the Aboriginal and Torres Strait Islander (ATSI) and the non‐Indigenous Australian populations. Additionally, the respondent's state of residence was considered, but the relative remoteness within the state of origin was not explored. If the geographical location had been further defined into rural and remote communities, it is possible that a significant association between location and FS would have been apparent. This may be important as both ATSI status and geographical location are both considered significant social determinants of FS in Australia. This is a limitation of the study and requires further research to ascertain the impact of location in relation to urban, rural and remote populations. Generalising these results across all populations is therefore cautioned.

The ABS data collection format is by face‐to‐face interview. The research presented in this study utilised an online form of data collection; however, this in itself can be considered a limitation. Online surveys as a mode of data collection preclude access of those without access to an Internet connection and people who cannot read written English. These are groups for whom the risk of FI has been shown to be higher than the general population.^14^ Nevertheless, it has been established that these vulnerable, hidden populations are hard to reach in general, even with traditional means of research such as interviews or paper‐based surveys employed by the ABS.^34^

## 5 CONCLUSION

Food insecurity remains an important issue across Australia. Our results indicate that certain groups, regardless of income, are particularly vulnerable to FI; these include younger Australians (particularly those aged 25‐34 years old), those with lower educational attainment and divorced or separated individuals. Government policy and community interventions that specifically target these “at risk” groups may assist to more effectively address the problem. Additionally, the use of the multi‐item measure is certainly worth considering in future studies and as a national indicator of FS in the Australian context.

## CONFLICT OF INTEREST

The authors declare no conflict of interest in connection with this article.
